# Physics of chewing in terrestrial mammals

**DOI:** 10.1038/srep43967

**Published:** 2017-03-07

**Authors:** Emmanuel Virot, Grace Ma, Christophe Clanet, Sunghwan Jung

**Affiliations:** 1 Emergent Complexity in Physical Systems Laboratory (ECPS), École Polytechnique Fédérale de Lausanne, CH 1015 Lausanne, Switzerland; 2 John A, Paulson School of Engineering and Applied Sciences, Harvard University, Cambridge, MA 02138, USA; 3 Department of Biomedical Engineering and Mechanics, Virginia Tech, Blacksburg, VA 24061, USA; 4 LadHyX, CNRS UMR 7646, École Polytechnique, 91128 Palaiseau, France; 5 PMMH, CNRS UMR 7636, ESPCI, 10 rue Vauquelin, 75005 Paris, France; 6 Center for Soft Matter and Biological Physics, Virginia Tech, Blacksburg, VA 24061, USA

## Abstract

Previous studies on chewing frequency across animal species have focused on finding a single universal scaling law. Controversy between the different models has been aroused without elucidating the variations in chewing frequency. In the present study we show that vigorous chewing is limited by the maximum force of muscle, so that the upper chewing frequency scales as the −1/3 power of body mass for large animals and as a constant frequency for small animals. On the other hand, gentle chewing to mix food uniformly without excess of saliva describes the lower limit of chewing frequency, scaling approximately as the −1/6 power of body mass. These physical constraints frame the −1/4 power law classically inferred from allometry of animal metabolic rates. All of our experimental data stay within these physical boundaries over six orders of magnitude of body mass regardless of food types.

In 1944, Erwin Schrödinger argued that organisms have evolved to avoid decay and to stay alive “by eating, drinking, breathing and (in the case of plants) assimilating”^1^. In the animal kingdom, eating is an essential activity of organisms from mycoplasmas to blue whales over twenty orders of magnitude in body size^2^. Food chewing has evolved over millions of years as a solution to increase digestive efficiency and achieve high levels of metabolic activities in terrestrial mammals (as compared to other vertebrates of similar masses), thereby setting the stage for endothermic temperature physiology and the fascinating diversification in mammals seen today^3^ (see examples of a cow, a horse, and sheep in Fig. 1).

Fortelius proposed that the volume of food per chew is proportional to the animal mass and that the food per unit time is proportional to the metabolic rate^4^, which scales as the 3/4 power of body mass according to Kleiber’s law^5^,^6^,^7^. As a consequence, the chewing frequency should be proportional to the −1/4 power of body mass (*Mf*_chew_ ~ *M*^3/4^). This model was supported by experimental observations of *f*_chew_ ~ *M*^−0.20^ 4. Later, Druzinsky observed a different scaling *f*_chew_ ~ *M*^−0.13^ by including small animals over three orders of magnitude in body mass, and concluded that the chewing frequency might not directly be related to the metabolic rate^8^.

Quite recently, Gerstner *et al*. have highlighted that all previous theoretical models have failed to describe correctly the contemporary data of chewing frequencies, which are midway between the previous two, i.e. *f*_chew_ ~ *M*^−0.15^ in ref. 9. This scaling seems to emerge from a scenario of optimal chewing where the chewing power is maximized (i.e. where the energy per chew is maximized while the time to chew is minimized). Based on Hill’s law, the muscle force and contraction speed are inversely correlated, so that the peak power is not simply achieved at the maximal force^10^. The peak power has been studied in the context of animal locomotion^11^,^12^, where the preferred speed of locomotion (*V*) lies between the 0.17 and 0.22 power of body mass. In analogy to the chewing motion, by assuming that the speed of muscle contraction is proportional to the motion speed and by assuming an amplitude of motion proportional to the jaw length (with *L*_jaw_ ~ *M*^1/3^ as precised in the present article), the chewing frequency *f*_chew_ ~ *V/L*_jaw_ is expected to lie between the −0.16 and −0.11 power of body mass.

Some recent studies also have suggested that the chewing frequency could match the jaw’s natural resonance frequency using the analogy of a pendulum (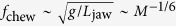; see e.g. refs 13,14 for primates and dogs). However, a gravity-driven chewing model is known to be biomechanically unrealistic regardless of the best fit to experimental observations^14^.

In summary, previous studies on chewing frequency have focused only on finding a single scaling; *f*_chew_ ~ *M*^−0.20^ for large animals^4^, *f*_chew_ ~ *M*^−0.13^ after including small animals^8^, *f*_chew_ ~ *M*^−0.15^ for the largest data-set^9^ and finally *f*_chew_ ~ *M*^−1/6^ based on pendulum-type movement of jaws^13^,^14^. Also, frequency variations were considered as statistical noise or randomness, which has generated a variety of scaling laws and aroused controversy between different models. Therefore, in contrast to the previous studies predicting a single functional relation between the chewing frequency and animal weight, in this study we determine the range of frequencies where animals can chew their food.

## Results

### Experimental data of the chewing frequency

Measurements of chewing frequency are reported on Fig. 2 over six orders of magnitude of animal mass. Black circles denote data that we measured from Virginia Tech farms, boxed rectangles are data that we estimated from online sources (see Materials and Methods) and triangles are measurements reported by^8^,^9^,^13^,^14^,^15^. We denote carnivores, herbivores, and omnivores with red, green, and blue colors, respectively. In the following sections, we focus on the role of saliva and muscles to explain the observed discrepancies.

### The saliva limit

Saliva is essential to chew, taste, and digest food. It lubricates between the mouth and food contents and between food contents themselves. Also, saliva enhances taste and digestion through bio-chemical processes. Salivary flow rate is known to vary depending on situations. For example, saliva is secreted at a very low flow rate when animals sleep or rest. However, when the salivary glands are mechanically stimulated during chewing, the saliva flow rate significantly increases. Animals have four pairs of major salivary glands connected to the oral cavity.

Figure 3(a) shows the saliva flow rate of various animals previously measured in refs 16, 17, 18, 19, 20, 21, 22, 23, 24, 25, 26, 27, 28, 29, 30, 31. We found an approximate power law for the flow rate of saliva *Q* ~ (4.8 × 10^−6^ kg^1/6^/s) *M*^5/6^ (best fit with a 0.87 power, *r*^2^ = 0.90, *n* = 30, *p* < 0.0001, 95% confidence interval: 0.79 to 1.00, see Fig. 3(a)). To efficiently mix saliva with food, the total amount of secreted saliva should be on the same order of magnitude with food amount within two consecutive swallows (which may include several chewing cycles) and should not exceed it. Therefore, based on the assumption that the saliva amount over the chewing period is close to the volume of oral cavity, we have


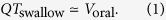


Here, *T*_swallow_ is the chewing time, equivalent to the number of chewing cycle times the inverse of chewing frequency, and *V*_oral_ is the volume of the oral cavity. The total number of chewing cycles before swallowing is measured to be 15.9 ± 5.1 over 21 primate species with four orders of magnitude of different body masses (this conclusion can be reached from the data measured by ref. 32). This number of cycles seems to be set by geometric relations: if we assume that the food is crunched into two pieces at every chewing motion, the number of chewing cycle should increase until the initial volume of food (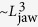) is ground to the size of upper esophageal sphincter for further digesting. Therefore, the total number of chewing cycles before swallowing is estimated as


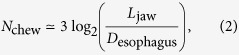


where *D*_esophagus_ is the diameter of the food pipe (esophagus). In this expression, both *L*_jaw_ and *D*_esophagus_ presumably scale isometrically with body mass, giving *N*_chew_ ≃ 10^1^ regardless of body mass. In case of humans^33^, *L*_jaw_/*D*_esophagus_ ≃ 20 and equation (2) becomes *N*_chew_ ≃ 13, which is close to the observations in primate species. This approach only gives the order of magnitude, further details are provided in ref. 34.

*V*_oral_ is the volume of the oral cavity, assumed to scale as the cube of jaw length (*V*_oral_ ≃ 4π(*L*_jaw_/2)^3^/3). The jaw length *L*_jaw_ is found to be *L*_jaw_ ≃ (5.0 × 10^−2^ m/kg^1/3^) *M*^1/3^ (best fit with a 0.37 power, *r*^*2*^ = 0.92, *n* = 95, *p* < 0.0001, 95% confidence interval: 0.35 to 0.40, see Fig. 3(b)). Therefore, the chewing frequency for saliva mixing verifies


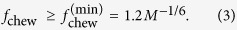


In Fig. 2, all data stand above this limit, which supports the validation of our model based on saliva mixing. Also, the exponent −1/6 is the same as a previously proposed model of pendulum-type chewing^14^, but based on different physics (our model is independent of gravity and head rotation).

### The muscle limits

The highest frequency of food chewing is presumably related to maximal muscle performance. Rhythmic chewing motion is modeled as a spring-like oscillation operated by the masseter muscles (Fig. 4). Based on ref. 14, the natural frequency of chewing for primates can be expressed as


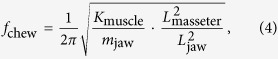


where the masseter lever distance, *L*_masseter_, is defined as the length between the masseter muscle and the jaw joint. Ross *et al*.^14^ showed that the masseter lever distance is about a half of the jaw length. First, we assume the jaw mass (*m*_jaw_) to be *ρ*_tissue_*V*_oral_ with *ρ*_tissue_ ≃ 10^3^ kg/m^3^ and 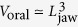, and the spring constant (*K*_muscle_) to be *F*_muscle_/(*L*_jaw_/2). Here, *L*_jaw_ ≃ (5.0×10^−2^ m/kg^1/3^) *M*^1/3^ as shown in the previous section. The maximum muscle force 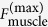 is proportional to the physiologic cross-sectional area (abbreviated as PCSA) of jaw muscle *A*_muscle_ ≃ (3.9×10^−4^ m^2^/kg^2/3^)*M*^2/3^ (best fit with a 0.73 power, *r*^2^ = 0.71, *n* = 91, *p* < 0.0001, 95% confidence interval: 0.63 to 0.82, see Fig. 3(c)). Also, the maximum muscle force per unit area, 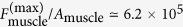 N/m^2^ is used^35^.

Finally, by combining all of the above values and relations, the chewing frequency verifies


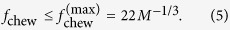


In addition, muscles are intrinsically limited in terms of contraction speed. Muscles typically consist of sarcomeres in series, of individual length *l*_s_ ≃ 2.5 *μ*m, all being shortened at the same speed (with ATP hydrolysis), and the maximal contraction speed relative to length should be essentially independent of body size: *v*_s_ ≃ 19 *μ*m/sarcomere/s^36^,^37^,^38^. Therefore we can assume that the frequency of jaw muscles also verifies


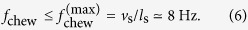


This intrinsic frequency presumably sets the upper limit of chewing frequency for small animals as observed in Fig. 2. For large animals heavier than 20 kg, the scaling of equation (5) prevails.

## Discussion

In contrast to the previous studies predicting a single scaling for the chewing frequency, here we have determined the range of chewing frequencies where terrestrial mammals can chew their food. Figure 2 shows that chewing behaviors are described by our proposed physical limits. The upper chewing frequency seems essentially limited by muscular actuation, and the lower chewing frequency is limited by mixing food with the right amount of saliva (i.e. without unnecessary excess) during a finite number of chews before swallowing.

The variations of chewing frequency in Fig. 2 could be primarily due to the type of food^14^,^31^,^39^. The upper limit in frequency derived in equation (5) is independent of the food type by essence. It can be considered as the inertial limit of the jaw motion. To take into account the role of food elasticity, we assume that the chewing power *P*_max_ developed by an animal to granulate food scales as its metabolic rate. Then, we postulate that this power is proportional to *EA*_dental_*f*_chew_*L*_jaw_, where *E* is the elastic modulus of the food and *A*_dental_ is the dental occlusion area, scaled isometrically with body mass (see ref. 32 and its references). As a consequence, we find *f*_chew_ ~ *E*^−1^*M*^−1/4^. This contribution is needed when the food rigidity *EL*_jaw_ is greater than the muscle rigidity *K*_muscle_. In case of humans, we have *K*_muscle_ ≃ 10^6^ N/m, thus the inertial model is valid when food elastic modulus does not exceed 10 MPa. Also, for large animals, chewing frequency is less affected by the food properties since their muscle rigidity is significantly larger than the food elasticity (*K*_muscle_ ~ *M*^1/3^).

In summary, the domain of chewing frequency is limited by several inequalities, not by a single power law. We find that chewing becomes an irrelevant mechanism if the minimal frequency required by efficient saliva mixing (~*M*^−1/6^) is higher than the maximal frequency at which muscles can be actuated, i.e. for animals heavier than 10^7^ kg or lighter than 10^−5^ kg. Therefore our work may also contribute to understanding why we do not observe terrestrial mammals as heavy as the mega sauropods (dinosaurs extinct approximately 100 millions years ago) of mass ~100 tons^40^,^41^,^42^, because their chewing frequencies would be presumably confined by the inertial and saliva-based limits in a small frequency range (0.2–0.5 Hz). Similarly, one cannot find any terrestrial mammal approaching the smallest weight limit, since the lightest contemporary mammal (Etruscan shrew) weighs about 1 g.

More generally, the upper limit for jaw oscillation frequency could be tested on rumination or even teeth-chattering. For future work, it would be interesting to consider how the chopping of soft and tough food by “our” teeth (which by itself requires energy) affects the physical limits of the chewing frequency.

## Materials and Methods

This study was carried out during the regular feeding times and animals were weighted during the maintenance period with the consents of farm managers. This study plan was discussed with, and approved by the Institutional Animal Care & Use Committee (IACUC) of Virginia Tech. All experiments presented in this manuscript were performed in accordance with relevant guidelines and regulations.

### Study subjects

**Figure 1:**
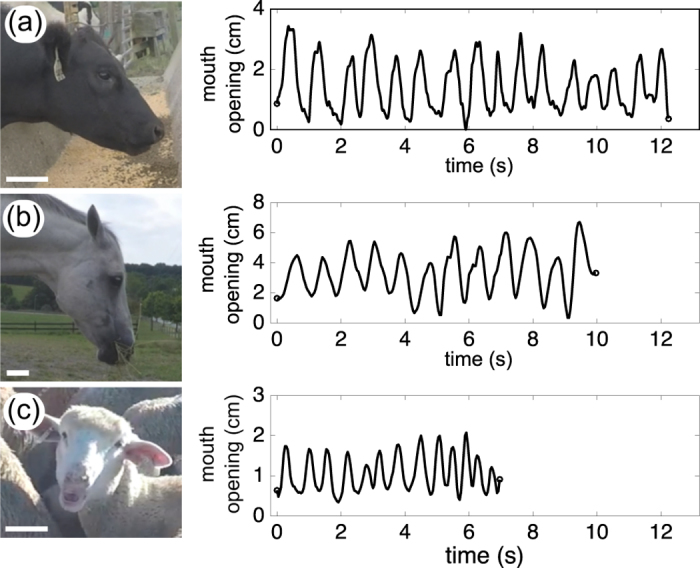
Time series of mouth opening in case of (**a**) cow, *M* = 427 kg, (**b**) horse, *M* = 476 kg and (**c**) sheep, *M* = 31 kg (see supplementary videos). The recordings start at the entrance of food in the mouth and stop at the first swallow of the animal. The scale bar indicates 10 cm.

**Figure 2:**
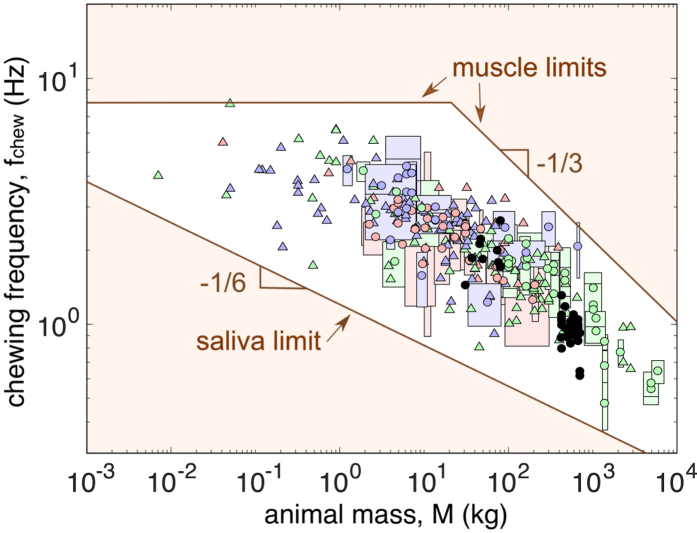
Plot of the chewing frequencies as a function of animal mass. Our own data come from 46 recordings in farms and from 86 online videos, denoted by circles. We draw the uncertainty boxes for online videos (see Materials and Methods). In addition, the data were supplemented by measurements reported in refs 8, 9 and 13, 14, 15, denoted by triangles. The color code is as follows: carnivores (red), herbivores (black for field data and green for other data), and omnivores (blue). All data are bounded by physical limits based on saliva and muscle. The upper limits are *f*_chew_ = 8 Hz for small animals and *f*_chew_ = 22 *M*^−1/3^ for large animals, whereas the lower limit is *f*_chew_ = 1.2 *M*^−1/6^.

**Figure 3:**
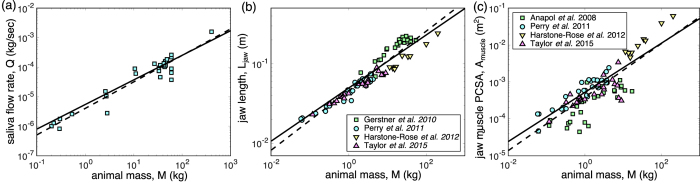
(**a**) Relation between saliva flow rate and animal mass. Data are obtained from^16^,^17^,^18^,^19^,^20^,^21^,^22^,^23^,^24^,^25^,^26^,^27^,^28^,^29^,^30^,^31^. The dashed line is the best fit with a 0.87 power, whereas the solid line is a 5/6 power as our assumption. (**b**) Relation between jaw length and animal mass. Data are obtained from^13^,^43^,^44^,^45^. The dashed line is the best fit with a 0.37 power, whereas the solid line is a 1/3 power as our assumption. (**c**) The relation between jaw muscle physiologic cross-sectional area (PCSA) and animal mass. Data are obtained from^43^,^44^,^45^,^46^. The dashed line is the best fit with a 0.73 power, whereas the solid line is a 2/3 power as our assumption.

**Figure 4:**
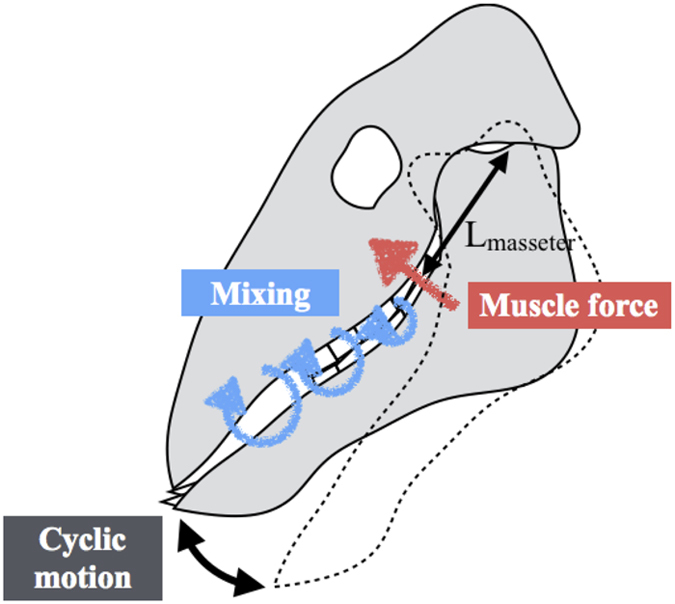
Drawing of the chewing motion. The masseter muscle is the main muscle involved in mastication, and the distance between the masseter muscle and the jaw joint is denoted as *L*_masseter_. The food is mixed with saliva, secreted by salivary glands.

Cows (*Bos taurus*), horses (*Equus caballus*) and sheep (*Ovis aries*) at Virginia Tech farms were chosen as subjects (Fig. 1 and supplementary videos). These animals were raised in good health and their body masses were measured within one month after recording chewing motion. A total of twenty animals were used for the analysis (nine cows, three horses and eight sheep). Individual animals were fed with daily food by their farm managers (cows and sheep with grain, and horses with dry hay). Then, chewing sequences were videotaped using two GoPro cameras at 120 fps. The chewing motion of these animals was analyzed from frame-by-frame image sequences. We excluded the chewing motion while animals were collecting or ruminating food. A chewing period was measured by the time interval between consecutive jaw closing moments, and the chewing frequency, *f*_chew_, is defined as the inverse of this chewing period. At least five chewing cycles were analyzed for individual animals.

In addition to these field measurements, we collected 86 videos of animals chewing food from online databases. We selected videos based on clear oscillatory chewing motions of animal. We paid special attention to finding animal species not locally accessible. Also, to get reliable statistics, the selected videos contain at least three cyclic chewing motions of each animal without a break. We determined a range of animal body mass from literature and encyclopedia. All the videos and the range of body mass are listed in the Tables S1, S2 and S3 (see electronic supplementary material).

## Additional Information

**How to cite this article**: Virot, E. *et al*. Physics of chewing in terrestrial mammals. *Sci. Rep.* **7**, 43967; doi: 10.1038/srep43967 (2017).

**Publisher's note:** Springer Nature remains neutral with regard to jurisdictional claims in published maps and institutional affiliations.

## Supplementary Material

Supplementary Information

Supplementary Video2

Supplementary Video3

Supplementary Video4
