# Supplementary material for: Physics of chewing in terrestrial mammals
Source: Sci Rep. 2017 Mar 7;7:43967. doi: 10.1038/srep43967 (PMC5339823; doi:10.1038/srep43967)
Supplement: Supplementary Information [file srep43967-s1.pdf]

Supplementary material for ”Physics of chewing in terrestrial mammals”

Emmanuel Virot[1,2,+], Grace Ma[3], Christophe Clanet[4,5,+], and Sunghwan Jung[3,6,+,*]

[1]Emergent Complexity in Physical Systems Laboratory (ECPS), École Polytechnique Fédérale de Lausanne, CH 1015 Lausanne, Switzerland
[2]John A, Paulson School of Engineering and Applied Sciences, Harvard University, Cambridge, MA 02138, USA
[3]Department of Biomedical Engineering and Mechanics, Virginia Tech, Blacksburg, VA 24061, USA
[4]LadHyX, CNRS UMR 7646, École Polytechnique, 91128 Palaiseau, France
[5]PMMH, CNRS UMR 7636, ESPCI, 10 rue Vauquelin, 75005 Paris, France
[6]Center for Soft Matter and Biological Physics, Virginia Tech, Blacksburg, VA 24061, USA
[+]these authors contributed equally to this work
[*]corresponding.sunnyjsh@vt.edu

The online videos that we have used are listed in the tables below (table S1, table S2 and table S3).

Animal	Youtube link	frequency ± std (Hz)	weight range (kg)	reference for the estimation of the weight
Chihuahua	v=lIOwgbyKi4M	2.53 ± 0.44	0.91 - 2.72	S.L. Gerstenfeld, J.L. Schultz, ASPCA Complete Guide to Dogs (1999)
Opossum	v=gkofwx2liHM	3.08 ± 0.32	1.30 - 5.00	R.M. Nowak, Walker's Mammals of the World (1999)
Opossum	v=Pv0AJWZr4h4	2.55 ± 0.25	1.30 - 5.00	R.M. Nowak, Walker's Mammals of the World (1999)
Opossum	v=VWwNkIRGnRk	3.18 ± 0.93	1.30 - 5.00	R.M. Nowak, Walker's Mammals of the World (1999)
Pomeranian	v=wxXSybfv-ss	2.24 ± 0.34	1.36 - 3.18	S.L. Gerstenfeld, J.L. Schultz, ASPCA Complete Guide to Dogs (1999)
Cat	v=7srW-W3FLiE	2.18 ± 0.17	4.00 - 5.00	M.Y. Mattern, D.A. McLennan, Phylogeny and Speciation of Felids, Cladistics 16 (2000)
Dachshund	v=rcrHj_EDf-s	2.66 ± 0.28	4.08 - 4.99	S.L. Gerstenfeld, J.L. Schultz, ASPCA Complete Guide to Dogs (1999)
Tasmanian Devil	v=MKbMGe7Cy1I	2.43 ± 0.29	5.5 - 11.8	R.M. Nowak, Walker's Mammals of the World (1999)
Tasmanian Devil	v=pD9xQTKkXTw	2.41 ± 0.33	5.5 - 11.8	R.M. Nowak, Walker's Mammals of the World (1999)
Jack Russell Terrier	v=8QfatAs8FwE	2.87 ± 0.45	6.30 - 6.50	D.C. Coile, Jack Russell Terriers (2000)
Shih-Tzu	v=I1Knt4e_Hzg	2.09 ± 0.25	6.35 - 8.16	S.L. Gerstenfeld, J.L. Schultz, ASPCA Complete Guide to Dogs (1999)
Beagle	v=h9wbed3MAN4	2.44 ± 0.65	8.2 - 13.6	S.L. Gerstenfeld, J.L. Schultz, ASPCA Complete Guide to Dogs (1999)
Beagle	v=vvh7n1kUqQw	2.94 ± 2.06	8.2 - 13.6	S.L. Gerstenfeld, J.L. Schultz, ASPCA Complete Guide to Dogs (1999)
Otter	v=2msCA_3FuII	2.93 ± 0.38	9.1 - 12.7	R. Link, Living with Wildlife in the Pacific Northwest (2005)
Bobcat	v=oq4_eoEFYDU	1.74 ± 0.56	9.8 - 10.6	V.T. Sparano, Complete Outdoors Encyclopedia (1998)
Cocker Spaniel	v=DLTINXElyCc	2.01 ± 0.25	11.8 - 15.4	S.L. Gerstenfeld, J.L. Schultz, ASPCA Complete Guide to Dogs (1999)
Dingo	v=5Qx549wFIk0	2.01 ± 0.57	13.0 - 20.0	L. Boitani, Simon & Schuster's Guide to Mammals (1984)
Border Collie	v=ywB_H-i949c	2.48 ± 0.43	15.9 - 25.0	S.L. Gerstenfeld, J.L. Schultz, ASPCA Complete Guide to Dogs (1999)
Border Collie	v=La1oc0fipt8	2.70 ± 0.32	15.9 - 25.0	S.L. Gerstenfeld, J.L. Schultz, ASPCA Complete Guide to Dogs (1999)
Husky	v=js_Rgh-l2jo	2.24 ± 0.60	15.9 - 27.2	S.L. Gerstenfeld, J.L. Schultz, ASPCA Complete Guide to Dogs (1999)
Dalmatian	v=I1Knt4e_Hzg	2.21 ± 0.55	16.0 - 32.0	S.L. Gerstenfeld, J.L. Schultz, ASPCA Complete Guide to Dogs (1999)
Dalmatian	v=JsAeUfjC5WE	2.81 ± 0.41	16.0 - 32.0	S.L. Gerstenfeld, J.L. Schultz, ASPCA Complete Guide to Dogs (1999)
Lynx	v=jmba6EWIdT8	1.56 ± 0.39	16.8 - 17.9	M. Sunquist, F. Sunquist, Wild Cats of the World (2002)
Golden Retriever	v=DaxkI_75c-8	2.32 ± 0.21	25.0 - 34.0	S.L. Gerstenfeld, J.L. Schultz, ASPCA Complete Guide to Dogs (1999)
Golden Retriever	v=x1C3vFOKEWM	2.52 ± 0.32	25.0 - 34.0	S.L. Gerstenfeld, J.L. Schultz, ASPCA Complete Guide to Dogs (1999)
Golden Retriever	v=9ZrgxMmkqg0	2.47 ± 0.17	25.0 - 34.0	S.L. Gerstenfeld, J.L. Schultz, ASPCA Complete Guide to Dogs (1999)
German Shepherd	v=m4C7gmMI5_E	1.80 ± 0.22	29.5 - 43.1	S.L. Gerstenfeld, J.L. Schultz, ASPCA Complete Guide to Dogs (1999)
Wolf	v=iYpkwqBJVs0	2.42 ± 0.49	31.3 - 40.4	B.H. Lopez, Of Wolves and Men (1978)
Jaguar	v=m9wC3xQK8h0	1.71 ± 0.28	36 - 158	R.M. Nowak, Walker's Mammals of the World (1999)
Jaguar	v=Br9–Sv4AdQ	1.53 ± 0.33	36 - 158	R.M. Nowak, Walker's Mammals of the World (1999)
Great Dane	v=c-FkLRG8lpI	1.69 ± 0.41	45.4 - 59.0	S.L. Gerstenfeld, J.L. Schultz, ASPCA Complete Guide to Dogs (1999)
English Mastiff	v=m-OasV9LCxE	1.49 ± 0.27	77.0 - 91.0	Guinness Book of World Records (1991)
American Mastiff	v=6-fFebLWcDo	1.98 ± 0.29	77.0 - 91.0	Guinness Book of World Records (1991)
Tiger	v=IoWXoB5ao8w	1.24 ± 0.43	180 - 306	R.M. Nowak, Walker's Mammals of the World (1999)
Lion	v=NpCmoHFhZSw	1.43 ± 0.41	272 - 313	K. Nowell, P. Jackson, Wild cats (1996)

Table 1. Carnivores. Information relative to the online Youtube videos (the general structure of the link is: www.youtube.com/watch?v=...).

Animal	Youtube link	frequency ± std (Hz)	weight range (kg)	reference for the estimation of the weight
Bunny	v=A9HV5O8Un6k	4.18 ± 0.38	0.91 - 1.13	Fur from Rabbits - A Collection of Articles
Rabbit	v=O-X1VtzmY00	2.77 ± 0.44	1.81 - 4.08	Nutrient Requirements of Rabbits (1966)
Red Panda*	v=Yp1xqOpYGnE	3.43 ± 0.65	3.18 - 6.35	D. Burnie, D.E. Wilson, Animal: The Definitive Visual Guide to the World's Wildlife (2005)
Sloth	v=xiE5AQHKj_Y	1.78 ± 0.37	3.63 - 4.99	F.B. Golley, E. Medina, Tropical Ecological Systems (1975)
Koala	v=AjFepCUx5Us	2.95 ± 0.88	4.0 - 15.0	R. Nowak, R. Walker's Marsupials of the World (2005)
Deer	v=2q91dUtDYkU	2.10 ± 0.24	45.3 - 68.0	S. Gallina, H. Lopez Arevalo, Odocoileus virginianus (2008)
Panda*	v=kCJujDysSPo	1.82 ± 0.32	70 - 160	G. Brown, Great Bear Almanac (1996)
Panda*	v=k-TFsd4768U	2.21 ± 0.32	70 - 160	G. Brown, Great Bear Almanac (1996)
Panda*	v=PMgN6-CUoLQ	1.69 ± 0.42	70 - 160	G. Brown, Great Bear Almanac (1996)
Panda*	v=4n0xNbfJLR8	1.74 ± 0.35	70 - 160	G. Brown, Great Bear Almanac (1996)
Mountain Goat	v=HvkVLEm18iI	1.79 ± 0.20	80 - 100	A. Ganeri, North America's Most Amazing Animals (2008)
Mountain Gorilla	v=6OnOrp4gcwQ	1.41 ± 0.10	100 - 195	G. Wood The Guinness Book of Animal Facts and Feats (1983)
Gorilla	v=FCADw-eS5_s	1.82 ± 0.45	102 - 147	P. Miller, Patricia Gorillas (1997)
Llama	v=QMwRo5ad19w	1.70 ± 0.34	110 - 120	R.B. Land, D.W. Robinson, Genetics of Reproduction in Sheep
Shetland Pony	v=hP05BgYyz_g	1.67 ± 0.29	180 - 270	S. Green, The Shetland Pony (2012)
Pony	v=N8go26JXgKc	1.73 ± 0.22	250 - 300	J. L. Falvey, An Introduction to Working Animals (1985)
Pony	v=oo2Wh20Iyf4	1.63 ± 0.18	250 - 300	J. L. Falvey, An Introduction to Working Animals (1985)
Pony	v=kXBMU9gXvjs	1.31 ± 0.26	250 - 300	J. L. Falvey, An Introduction to Working Animals (1985)
Horse	v=n-8YRlLHoM4	1.49 ± 0.20	380 - 550	M. Bongianni, Simon & Schuster's Guide to Horses and Ponies (1987)
Arabian Horse	v=gic0W9tUpm0	1.60 ± 0.44	408 - 544	M. Bongianni, Simon & Schuster's Guide to Horses and Ponies (1987)
Rhino	v=EBOU9j0BcmQ	0.93 ± 0.10	800 - 1200	M. Fitzpatrick, K. Armstrong, South Africa, Lesotho & Swaziland (2006)
Rhino	v=-sEoOrJGTRY	1.05 ± 0.07	800 - 1200	M. Fitzpatrick, K. Armstrong, South Africa, Lesotho & Swaziland (2006)
Giraffe	v=wiuNqeKdzyU	1.39 ± 0.22	828 - 1192	J.D. Skinner, R.H.M. Smithers, The mammals of the southern African subregion (1990)
Giraffe	v=pk-vOfQNMuk	1.19 ± 0.15	828 - 1192	J.D. Skinner, R.H.M. Smithers, The mammals of the southern African subregion (1990)
Giraffe	v=tnBkhcRlixQ	1.13 ± 0.32	828 - 1192	J.D. Skinner, R.H.M. Smithers, The mammals of the southern African subregion (1990)
Hippo	v=OxNXQ9bzYTo	0.67 ± 0.11	1300 - 1500	R. N. Owen-Smith Megaherbivores: The Influence of Very Large Body Size on Ecology (1995)
Hippo	v=wq_xVYMvVa8	0.47 ± 0.10	1300 - 1500	R. N. Owen-Smith Megaherbivores: The Influence of Very Large Body Size on Ecology (1995)
Hippo	v=86vpgweBiGI	0.84 ± 0.06	1300 - 1500	R. N. Owen-Smith Megaherbivores: The Influence of Very Large Body Size on Ecology (1995)
White Rhino	v=aUI744klo3Y	0.76 ± 0.10	1600 - 2400	J. D. Skinner, C. T. Chimimba, The Mammals Of The Southern African Subregion (2005)
Elephant	v=4LivRehYGkE	0.54 ± 0.07	3000 - 5000	J. Shoshani, Elephants: Majestic Creatures of the Wild (2000)
Elephant	v=6umRDWnfivI	0.57 ± 0.06	3000 - 5000	J. Shoshani, Elephants: Majestic Creatures of the Wild (2000)
African Elephant	v=M7RA-hFz6s0	0.64 ± 0.07	4000 - 7000	J. Shoshani, Elephants: Majestic Creatures of the Wild (2000)

Table 2. Herbivores. Information relative to the online Youtube videos (the general structure of the link is: www.youtube.com/watch?v=...). *Strictly speaking, pandas have a digestive system of carnivores and food habits of herbivores.

Animal	Youtube link	frequency $\pm$ std (Hz)	weight range (kg)	reference for the estimation of the weight
Chinchilla	v=fQGxee2yLgA	4.24 ± 0.59	0.41 - 0.42	A.E. Spotorno et al., Mammalian Species 758
Skunk	v=TteE3dKirac	4.08 ± 0.48	1.80 - 5.50	G. Feldhamer, B. Thompson, J. Chapman, Wild Mammals of North America (2003)
Skunk	v=9-myK_W4HFs	3.39 ± 0.79	1.80 - 5.50	G. Feldhamer, B. Thompson, J. Chapman, Wild Mammals of North America (2003)
Coati	v=6Iit35gGtUY	2.83 ± 0.63	2.00 - 8.00	K. M. Helgen et al., Small Carnivore Conservation 41 (2009)
Coati	v=6Iit35gGtUY	3.91 ± 0.54	2.00 - 8.00	K. M. Helgen et al., Small Carnivore Conservation 41 (2009)
Baby Raccoon	v=DjAo_9eSPWI	4.36 ± 1.44	3.50 - 9.00	S.I. Zeveloff, Raccoons: A Natural History (2002)
Raccoon	v=PxaW5JSyijY	2.93 ± 0.55	3.50 - 9.00	S.I. Zeveloff, Raccoons: A Natural History (2002)
Raccoon	v=0jwGq0I4xEY	4.04 ± 0.65	3.50 - 9.00	S.I. Zeveloff, Raccoons: A Natural History (2002)
Raccoon	v=RavcwqH0Ou0	3.42 ± 0.40	3.50 - 9.00	S.I. Zeveloff, Raccoons: A Natural History (2002)
Rock Hyrax	v=iKJcZ3e8E6Y	3.63 ± 0.34	5.00 - 6.00	T. Parkinson, M. Phillips, W. Gourlay, Kenya (2006)
Coyote	v=qLH2qFx-jB4	2.97 ± 0.35	12.5 - 14.0	M. Bekoff M., Mammalian Species 79 (1977)
Coyote	v=VACtjNhShFE	2.62 ± 0.29	12.5 - 14.0	M. Bekoff M., Mammalian Species 79 (1977)
Coyote	v=Uv58Kk9BsxM	2.63 ± 0.19	12.5 - 14.0	M. Bekoff M., Mammalian Species 79 (1977)
Pig	v=DyafXlWjNyc	2.46 ± 0.40	30.0 - 33.0	M.M. Swindle, A.C. Smith, Swine in the Laboratory (2016)
Baby Pig	v=7qpETkG3vjA	2.45 ± 0.41	30.0 - 33.0	M.M. Swindle, A.C. Smith, Swine in the Laboratory (2016)
Sumatran Orangutan	v=APt4b3FWRnE	1.21 ± 0.24	37.0 - 75.0	C.P. Groves, Mammalian Species 4 (1971)
Human	v=KQfDBZI1MOc	1.71 ± 0.42	80.6 - 80.8	S.C. Walpole et al., The weight of nations: an estimation of adult human biomass (2012)
Bear	v=TiHmBLhDJY8	1.94 ± 0.24	115 - 360	M. Pasitschniak-Arts, Mammalian Species (1993)
Russian Bear	v=SHaZqBrri20	2.07 ± 0.53	650 - 700	M. Pasitschniak-Arts, Mammalian Species (1993)

Table 3. Omnivores. Information relative to the online Youtube videos (the general structure of the link is: www.youtube.com/watch?v=...).
